# Text-Mining to Identify Gene Sets Involved in Biocorrosion by Sulfate-Reducing Bacteria: A Semi-Automated Workflow

**DOI:** 10.3390/microorganisms11010119

**Published:** 2023-01-03

**Authors:** Payal Thakur, Mathew O. Alaba, Shailabh Rauniyar, Ram Nageena Singh, Priya Saxena, Alain Bomgni, Etienne Z. Gnimpieba, Carol Lushbough, Kian Mau Goh, Rajesh Kumar Sani

**Affiliations:** 1 Department of Chemical and Biological Engineering, South Dakota School of Mines and Technology, Rapid City, SD 57701, USA; 2 Data Driven Material Discovery Center for Bioengineering Innovation, South Dakota School of Mines and Technology, Rapid City, SD 57701, USA; 3 Department of Biomedical Engineering, University of South Dakota, Sioux Falls, SD 57069, USA; 4 2-Dimensional Materials for Biofilm Engineering, Science and Technology, South Dakota School of Mines and Technology, Rapid City, SD 57701, USA; 5 Faculty of Science, Universiti Teknologi Malaysia, Skudai 81310, Johor, Malaysia; 6 BuG ReMeDEE Consortium, South Dakota School of Mines and Technology, Rapid City, SD 57701, USA; 7 Composite and Nanocomposite Advanced Manufacturing Centre—Biomaterials, Rapid City, SD 57701, USA

**Keywords:** biocorrosion, sulfate-reducing bacteria, text mining, metal ion, sulfur metabolism

## Abstract

A significant amount of literature is available on biocorrosion, which makes manual extraction of crucial information such as genes and proteins a laborious task. Despite the fast growth of biology related corrosion studies, there is a limited number of gene collections relating to the corrosion process (biocorrosion). Text mining offers a potential solution by automatically extracting the essential information from unstructured text. We present a text mining workflow that extracts biocorrosion associated genes/proteins in sulfate-reducing bacteria (SRB) from literature databases (e.g., PubMed and PMC). This semi-automatic workflow is built with the Named Entity Recognition (NER) method and Convolutional Neural Network (CNN) model. With PubMed and PMCID as inputs, the workflow identified 227 genes belonging to several *Desulfovibrio* species. To validate their functions, Gene Ontology (GO) enrichment and biological network analysis was performed using UniprotKB and STRING-DB, respectively. The GO analysis showed that metal ion binding, sulfur binding, and electron transport were among the principal molecular functions. Furthermore, the biological network analysis generated three interlinked clusters containing genes involved in metal ion binding, cellular respiration, and electron transfer, which suggests the involvement of the extracted gene set in biocorrosion. Finally, the dataset was validated through manual curation, yielding a similar set of genes as our workflow; among these, *hysB* and *hydA*, and *sat* and *dsrB* were identified as the metal ion binding and sulfur metabolism genes, respectively. The identified genes were mapped with the pangenome of 63 SRB genomes that yielded the distribution of these genes across 63 SRB based on the amino acid sequence similarity and were further categorized as core and accessory gene families. SRB’s role in biocorrosion involves the transfer of electrons from the metal surface via a hydrogen medium to the sulfate reduction pathway. Therefore, genes encoding hydrogenases and cytochromes might be participating in removing hydrogen from the metals through electron transfer. Moreover, the production of corrosive sulfide from the sulfur metabolism indirectly contributes to the localized pitting of the metals. After the corroboration of text mining results with SRB biocorrosion mechanisms, we suggest that the text mining framework could be utilized for genes/proteins extraction and significantly reduce the manual curation time.

## 1. Introduction

Corrosion is defined as the degradation of steel, iron, and concrete materials, affecting many industries globally, such as oil and gas, sewage, construction, and drinking water systems [1]. The advanced metal deterioration in the presence of microbes is termed microbial induced corrosion (MIC) or biocorrosion. MIC is a result of collaborative interactions between the metallic surface, corrosion byproducts, bacteria, and their corrosive metabolites. These include the production of hydrogen sulfide, ammonia, and some organic and inorganic acids [2]. Several MIC mechanisms have been described, including chemical MIC (CMIC) and electrical MIC (EMIC) [3]. Metal corrosion by the production of corrosive metabolites such as hydrogen sulfide is known as CMIC, whereas the corrosion involving direct electron transfer between bacteria and metal is known as EMIC [4]. MIC is not only the result of complex corrosion reactions, but also the result of a series of flawless events involved in the construction of a biofilm, the most intricate, highly organized, and successful mode of life for microorganisms. Biofilms consist of microbial communities attached to the metal surface, embedded in self-produced EPS, proteins, lipids, and extracellular DNA [5].

Microbes in their natural habitats live in synergistic mixed-culture biofilms. Biofilms are responsible for MIC as they can harbor high concentrations of corrosive agents underneath and harvest electrons from energetic metals [6]. SRB are a major class of anaerobic, biofilm-forming bacteria responsible for biocorrosion. These bacteria have enzymatic systems that participate in different corrosion process steps. First, their hydrogenases depolarize the metal surface, leading to its solubilization and generation of electrons. These electrons are then transferred to sulfate for its reduction to hydrogen sulfide, which influences further metal dissolution [7]. The corrosion mechanism by SRB, known as cathodic depolarization theory (CDT), was first explained in the year 1934 by Wolzogen Kuhr and van der Vlugt. The theory states that SRB catalyze the depolarization of the cathode by the metallic oxidation of hydrogen for its innate process of sulfate reduction. Moreover, the cathodic hydrogen consumption by SRB and its enzyme hydrogenases leads to the enhanced corrosion of the metal [8]. SRB acquires its energy by using sulfate as the final electron acceptor, followed by its ultimate reduction to hydrogen sulfide, dissimilatory sulfate reduction. The role of hydrogen sulfide and other metal sulfides has been reported in various research articles with context to MIC. For example, in 1971, King and Miller reported a positive shift in the corrosion potential when conductive iron sulfide film was formed on the metal surface. Moreover, persistent corrosion by metal sulfides can only occur in the presence of viable SRB populations. Another theory supporting the influence of hydrogen sulfide in biocorrosion was reported in 2009, known as BCSR (biocatalytic cathodic sulfate reduction). According to this theory, SRB form biofilms and experience enhanced sulfate reduction under anoxic conditions, augmenting biocorrosion reactions [2,3,4]. Other SRB corrosion mechanisms are also defined: A Volatile Phosphorous Compound (1983), Anodic Depolarization (1984), Fe-Binding Exopolymers (1995), Sulfide and Hydrogen-Induced Stress Corrosion Cracking (SCC) (1995), Sulfide (1998), and Three Stages Mechanism (2005) [9]. It is becoming evident that an enormous amount of literature is present in the context of MIC mechanisms in scientific databases. However, the increased number of publications makes it difficult for the researchers to keep up with the manual curation from the published corpora. Additionally, the curators face inter-annotator disagreement: as different curators may decipher a text differently than others [10]. Current developments in text mining (the method of obtaining relevant data from unstructured text) and machine learning (investigation of techniques that can be trained from a particular data-set and has decision-making capabilities with slight human involvement) have facilitated explanations to tricky questions [11].

Despite the fast-growing studies on corrosion (PubMed alone has more than 500 articles), there are limited collections of genes involved in the corrosion process; biocorrosion comprised 61 results in total at the time of our study. It is also becoming evident that there is no single dominant mechanism for biocorrosion, and experimental validation of all these theories can be arduous. Therefore, improved technology is required to identify genes and proteins of SRB participating in the biocorrosion process; it can help create relevant biological processes and other metabolic pathways associated with these proteins. Our present work examines PubMed and PMC databases that contain information about MIC caused by SRB using a text-mining approach. The text-mining tool utilized in this study is a semi-automatic workflow built on the Named Entity Recognition (NER) method and Convolutional Neural Network (CNN) model, which depends on text granulation and a constant learning algorithm as the input model [12]. Moreover, pangenome analysis was utilized to investigate the occurrence of these genes across 63 genomes of *Desulfovibrio*. Noting that the MIC is not caused by a single mechanism, but rather by a range of cellular mechanisms, for example sulfur metabolism, biofilm formation, and quorum sensing that can aggravate the corrosion process. Therefore, we performed protein–protein networks, GO enrichment, and pangenome analysis. This information can be used to reveal the role of these genes responsible for metal corrosion by *Desulfovibrio* species.

## 2. Materials and Methods

### 2.1. Data Retrieval

To obtain a set of publications focused on biocorrosion, we formulated the following PubMed and PMC query in April 2020: (Biocorrosion OR “Microbial Corrosion”) (Gene OR Protein) AND (Sulfate Reducing Bacteria OR “Sulfate Reducer” OR SRB).

### 2.2. Data Selection and Preprocessing

**Figure 1:**
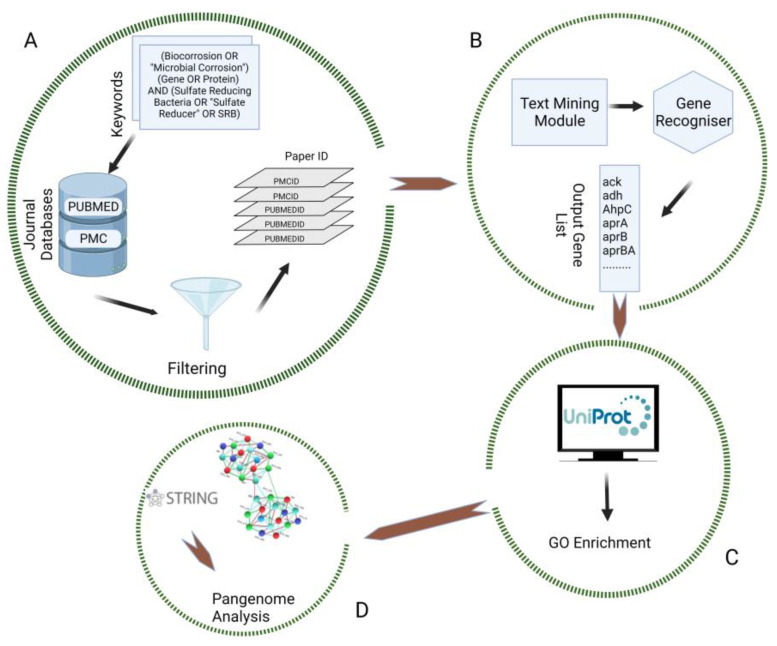
Text-mining workflow utilized to obtain relevant gene lists. (**A**) Query formulation to extract relevant research articles; (**B**) Gene mining from the literature; (**C**) Enrichment analysis of the gene sets; (**D**) Pangenome and PPI network analysis.

The search result titles and abstracts were computationally inspected for relevant keywords using a custom script. Articles that did not contain Sulfate, SRB, Protein, and Biocorrosion were excluded. Selected papers were fed into GenNER [12], a semi-automatic Named Entity Recognition (NER) text-mining workflow based on deep learning coupled with Convolutional Neural Network. GenNER was selected because it outperformed the existing gene NER model on prokaryote gene name entity recognition. For example, the retrieval of *Desulfovibrio vulgaris* Hildenborough (DvH) corrosion genes from the paper PMC4847118 using the gold standard tools from NCBI Pubtator yielded zero genes [12], but GenNER was able to retrieve all genes as described in our result (Supplementary Materials Table S1). This text-mining process was followed by retrieving relevant genes/proteins from the obtained articles employing a retrained custom model GenNER to extract the essential data automatically. Finally, the data extracted from GenNER was validated through manual curation of genes/proteins (Supplementary Materials Tables S2 and S3). Genes belonging to genera *Desulfovibrio*, *Desulfoglaeba*, *Desulfofacinum* and *Desulfotomaculum*, were retrieved by the combination of automated curation and manual curation (Figure 1). In addition, further analysis was conducted on the genes/proteins belonging to DvH.

### 2.3. GO Enrichment and Biological Network Building

The dataset obtained was biocurated using the UniProt database. This database separated the genes centered on their gene ontology (GO) terms, molecular function (MF), cellular component (CC), and biological process (BP). Furthermore, the genes were classified based on their involvement in transport activity, sulfur metabolism, cell adhesion, cell cycle and nucleic acid binding, cellular respiration, exopolymer synthesis, carbon, and cell energy metabolism. Finally, STRING-DB enrichment was performed to validate the acquired gene sets. Therefore, STRING-DB is a useful tool that provides protein–protein interaction (PPI) networks, which are crucial for the system-level interpretation of GO terms. Nodes denote the proteins, and the edges represent the interactions in the PPI network. In addition, the amalgamation of the acquired genes into a single cluster was performed by implementing the k-means clustering algorithm in STRING-DB. The network obtained from STRING-DB was subjected to enrichment analysis utilizing Cytoscape (version: 3.9.1). Furthermore, the enrichment table containing the p-values for every GO-term was processed with a multienrichjam R package (version: 0.0.65.900) and visualized with an enrichplot R package (version: 3.16).

### 2.4. Gene Set Distribution Analysis across the SRB Genome

To further improve our understanding of the obtained gene set, the pangenome developed by Shailabh et al. (under preparation) was employed to illustrate the distribution of gene sets throughout the genus *Desulfovibrio*. The pangenome consists of a set of 63 SRB genomes acquired from the National Center for Biotechnology Information (NCBI) and JGI Integrated microbial genomes and microbiomes (JGI/IMG). The pangenome workflow is available for use on the Google Colab platform. The detailed information of the geneset for the text mining was downloaded through the UniProt Retrieve/ID mapping. Subsequently, the FASTA files of the gene set were uploaded to the Google Colab (PPanGGOLiN) [13] to align the genes with the SRB pangenome. The pangenome follows the PPanGGOLiN pipeline to build pangenomes for large sets of prokaryotic genomes by utilizing a statistical technique and a graphical method that segregates the gene families into three major partitions: shell, cloud, and persistent. This pipeline uses N genes encoding proteins and their genomic neighborhood to develop a graphical representation where every gene family is represented by a node, and each edge signifies genetic contiguity (defined as the link between two neighboring genomes in the graph). The output file consisted of the corresponding gene families from pangenome, partition, and quality parameters in terms of percent identity (pident), e-value (expectation value) and bit score for each input gene. Consequently, the matching gene family matrix data was retrieved to generate the presence/absence matrix (P/A) for the input gene list (Supplementary Materials Table S4). In Table S4, rows and columns represent gene families and genomes, respectively, where the presence of a gene family is indicated by a value of 1 and its absence is denoted by a value of 0. A multivariate BMM (Bernoulli Mixture Model) was utilized to model the P/A matrix. Furthermore, the Expectation Maximization (EM) algorithm assessed the parameters of the matrix considering the constraints governed by the Markov Random Field (MRF). In accordance with the BMM algorithm, the closest partition was associated with each gene family.

## 3. Results and Discussions

### 3.1. Data Mining and Preprocessing

The query resulted in 292 publications, 21 in PubMed and 271 in PMC, at the time of our study (April 2020). Therefore, the search for relevant publications was narrowed to 51 research articles from both databases (PubMed-8; PMC-43). Using our text-mining tool, we extracted 516 genes from these 51 papers. After duplicate removal and manual curation, 227 genes were extracted belonging to genera *Desulfovibrio*, *Desulfofacinum*, *Desulfotomaculum*, and *Desulfoglaeba* (Supplementary Materials Table S2). The study focuses on DvH, as DvH has been considered as a model organism to explore MIC and energy metabolism in SRB [14,15]. Consequently, the search is centered on the genes belonging to DvH; 43 genes (Supplementary Materials Table S3) were extracted from previously curated 227 genes belonging to different *Desulfovibrio* species.

### 3.2. Gene Analysis Based on GO Terms

**Figure 2:**
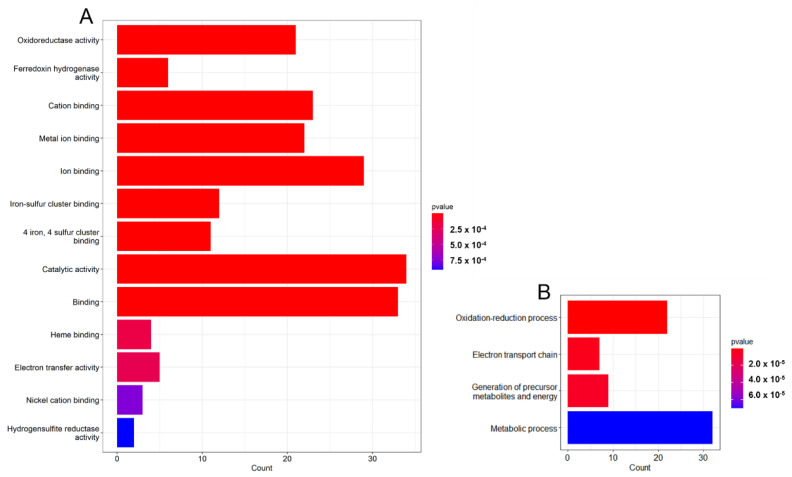
Functional enrichment analysis of GO terms (**A**) molecular function and (**B**) biological process. All the terms are statistically significant with a *p*-value < 0.05.

The functional annotation was performed using the UniProt database for the selected gene list. This allowed us to classify the gene list on the basis of gene ontology (GO) terms, biological process (BP), molecular function (MF), and cellular component (CC), where GO defines the characteristics of genes and gene products. The cellular component indicates a region of an active gene product in a cell, whereas the MF defines the basic activity of a gene product at the molecular level. Finally, the BP is described as the contribution of genes and gene products to the biological objective [16]. Figure 2 signifies the enriched GO terms in the molecular function and biological process category. As can be noted from Figure 2, all the enriched GO terms are statistically significant with a *p*-value < 0.05. The MF and BP plots show that the enriched terms belong to electron transport, ion binding (ATP binding), and metal ion binding pathways necessary for the growth and survival of bacteria. In addition, microbes depend on the oxidation/reduction processes for ATP generation and growth.

Bacterial exopolysaccharides might fluctuate in their capability to ionize and attach to metal ions. For instance, several interactions were observed between exopolymers and copper ions, which resulted in the copper concentration cell formation on the copper metal surface, promoting the deterioration of the underlying metal. Moreover, ATP plays a vital role in the energy metabolism of all living cells. The bacteria utilize the free energy released by the hydrolysis of the bonds between phosphate groups of ATP to develop and carry out cellular processes (e.g., sulfate reduction and biofilm formation) [14,17,18].

### 3.3. Network Building and Cluster Analysis

**Figure 3:**
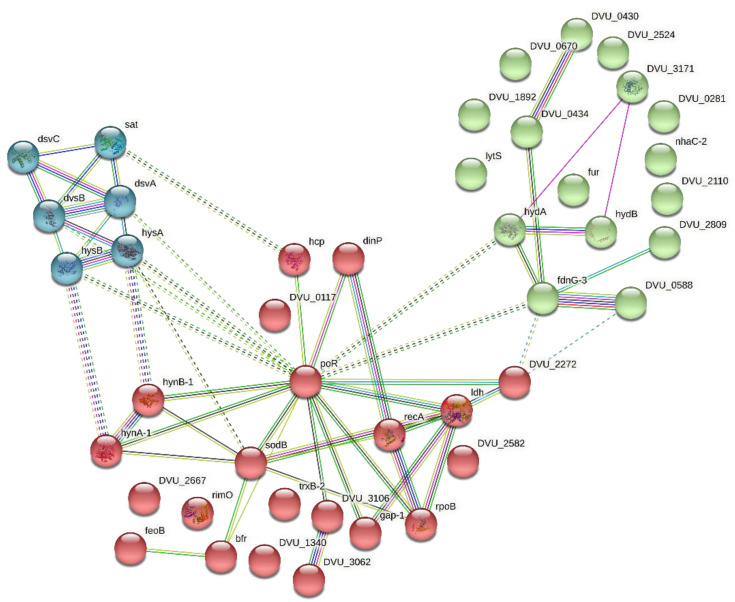
The PPI network demonstrates three cluster formations. Cluster 1 (red) shows the genes involved in metal ion binding and oxidoreductase activity. Cluster 2 (green) represents the genes participating in cellular respiration, oxidation-reduction activity, and electron transport. Lastly, cluster 3 (cyan) displays genes engaged in oxidoreductase, hydrogenase and hydrogensulfite reductase activity.

The PPI network is one of the most applicable network types to signify the functional association of the genes encoding proteins in a given genome [19]. Since there are multiple ways in which a protein can interact, a ‘functional association’ signifies the interaction of two proteins contributing towards a specific cellular process. Moreover, it can also be defined as the interaction of two proteins that can act distinctively within a single pathway. The STRING-DB is one of the various databases available online devoted to the organism-wide protein association networks [20,21]. STRING was utilized to build a PPI for the selected gene list with the objective of combining data that could recognize genes/proteins involved in MIC. The k-means clustering categorized the gene list into three clusters (Figure 3). Cluster 1 (red) comprised 21 genes, primarily participating in metal ion binding. Karn et al., 2020, reported that the binding of the metal ions with the biofilm matrix of the bacteria led to the formation of complexes that participated in the process of electron transfer, influencing the corrosion reactions [22]. Cluster 2 (green) consists of 16 genes associated with it, mainly involved in electron transfer and cellular respiration activities. Hamilton et al. (2003) formulated a unifying electron-transfer hypothesis of MIC, according to which electron transfer between the microorganism and the metal play a role in corrosion. The metabolic events of microorganisms produce insoluble products capable of accepting electrons from the base metal, creating a kinetically favored electron flow pathway from the metal acting as anode and sulfate as the final electron acceptor, in the case of SRB [23]. Moreover, the activity of direct electron transfer (DET) between the hydrogenases enzyme of SRB and the metal has also been reported to influence the corrosion of stainless steel [24]. Finally, cluster 3 (cyan) holds six genes participating in microbial and sulfur metabolism in various environments. Anaerobic metabolism combined with the reduction of sulfate to sulfide and oxidation of organic compounds aids SRB in obtaining energy for growth. SRB obtain energy for growth from anaerobic metabolism, combining the oxidation of organic substrates with the reduction of sulfate to sulfide [25]. Sulfur and its complexes have a high affinity for some common alloys and metals. Microbial metabolic processes may produce corrosive metabolites, such as sulfuric acid (H_2_SO_4_), organic acids, and organic or inorganic sulfides. Therefore, metabolic products make the surrounding environment corrosive for the common metals [26]. Moreover, the interaction between clusters 1, 2, and 3 indicates a strong correlation between metal ion binding, electron transport, and sulfur metabolism in driving corrosion reactions. For instance, the hydrogenases enzyme of SRB plays an active role in hydrogen metabolism, which plays a central part in energy generation for the microorganism. In the periplasm, hydrogenases oxidize the externally produced hydrogen (H_2_), releasing electrons. Now, the released electrons are captured by the c-type cytochrome network, channeling them through transmembrane complexes. Finally, these electrons are utilized to reduce sulfate and generate H_2_S, contributing to the sulfur metabolism of SRB. The biogenically produced sulfide binds with metal ions, forming metal–sulfide complexes that further influence metal dissolution [27]. Moreover, some SRB strains can directly accept electrons from the metal surface, encouraging MIC; this process of DET between SRB hydrogenases enzyme and metal surface offers a deep understanding of the electron transport mechanisms [24]. The involvement of these genes in stimulating biocorrosion is discussed in Section 3.5.

### 3.4. Gene Segregation Based on SRB-Pangenome Analysis

**Figure 4:**
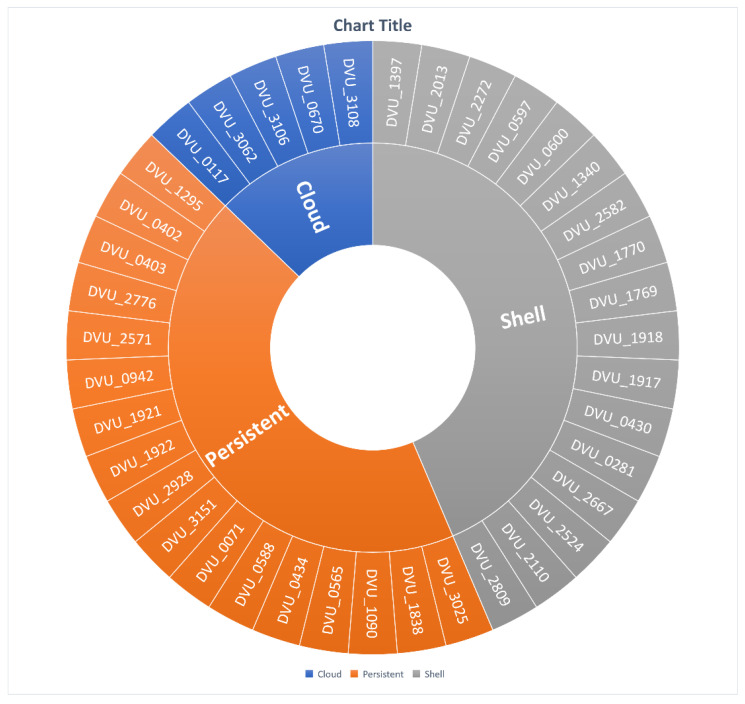
The figure represents the distribution of 39 genes obtained by text mining across 63 SRB pangenomes. Note: see Supplementary Materials Table S4 for more information on these genes.

Currently, researchers mostly use probabilistic models [28] and the Binomial Mixture Model [29,30] for pangenome analysis. PPanGGOLiN was utilized to categorize the genes based on a statistical method to classify them according to their occurrence in 63 SRB genomes. Unlike the existing approaches, PPanGGOLiN does not rely on frequencies to execute gene characterization, but is a combination of gene family occurrence patterns and topology of the pangenome graph. This graph-based approach effectively generates a compact structure which describes the genomic distinctiveness of a large number of strains. Based on the presence of the gene families across pangenome, the gene families can be divided into three main classes: (1) persistent, which includes gene families existing in nearly all genomes; (2) shell, comprising gene families appearing at moderate frequencies in the species; and (3) cloud, containing gene families existing in small frequencies in species [13]. In our study, the persistent category consisted of 17 genes, the shell comprised 17 genes, and the remaining 5 genes were a part of the cloud gene families (Figure 4).

#### 3.4.1. Persistent Gene Family

Analysis results showed that out of 17 genes (Supplementary Materials Table S4) from the persistent genome, eight genes are present in all 63 genomes, four genes in 62 genomes, and two genes in 61 genomes. The uniformly present eight genes were *dsvC*, *hynA-1*, *hynB1, feoB, fur, rimO, recA*, and *rpoB*. The gene *dsvC* encodes for the subunit gamma of dissimilatory-type sulfite reductase, an important enzyme for sulfate-reducing mechanism and energy generation in SRB [31]. Two genes, *hynA-1* and *hynB1*, encode for the large subunit and the small subunit of Periplasmic [NiFe] hydrogenase isozyme 1, respectively. Hydrogenases generate protons and electrons for energy requirements. [NiFe] hydrogenase is less sensitive to CO, NO, and NO^2−^ inhibition and plays an important role in the regulation of gene expression in different metal concentrations [32]. The genes *feoB* and *fur* are involved in iron uptake and regulation [33], and code for ferrous iron transport protein B and ferric uptake regulation protein, respectively. The gene *rimO* encodes for enzyme methylthiotransferase, adding a methylthio group to small ribosomal protein S12, and is involved in post-translational modification [34]. *recA* gene plays a significant role in cell survival by DNA repair mechanisms at different stresses, such as thermal or oxygen stress [35]. In contrast, *rpoB* encodes for the beta subunit of RNA polymerase and is involved in transcription.

Four genes, which include *dsvA*, *sat*, *dinP* and DVU0588, were present in 62 genomes in the persistent gene family category. The gene *dsvA*, which encodes for the alpha subunit of dissimilatory-type sulfite reductase, was missing in *Desulfovibrio magneticus* strain Maddingley MBC34. Genes such as sat codes for sulfate adenylyltransferase are missing in the genome of *Desulfovibrio* sp. G11, *dinP* encodes for DNA polymerase IV, and is missing in *Desulfovibrio legallii*. Gene DVU0588 encodes for the beta subunit of formate dehydrogenase, playing an important role in anaerobic metabolism [36]; it was found to be absent in *Desulfovibrio* sp. ZJ746. Two genes (*dsvB* and *poR*) in the persistent gene family were only shared by 61 genomes. Gene *dsvB* was absent in *Desulfovibrio magneticus* strain Maddingley MBC34 and *Desulfovibrio* sp. AS27yjCOA_33, while *poR* was found to be missing in genomes of *Desulfovibrio carbinoliphilus* subsp. *Oakridgensis* and *Desulfovibrio sulfodismutans*. These results suggest that *Desulfovibrio magneticus* strain Maddingley MBC34 does not have genes for alpha and beta subunits of dissimilatory-type sulfite reductase.

The *trxB*-2, which encodes for thioredoxin reductase, reduces thioredoxin, thereby regulating the intracellular redox potential in anaerobic microbes [37]. *TrxB*-2 was absent in the genomes of *Desulfovibrio bizertensis* MKS_re-assembly, *Desulfovibrio ferrophilus*, and *Desulfovibrio vietnamensis* DSM10520. Six out of 63 genomes were lacking in gene *gap-1*, which codes for glyceraldehyde-3-phosphate dehydrogenase. These six genomes are *Desulfovibrio bizertensis* MKS_re-assembly, *Desulfovibrio desulfuricans* ND132, *Desulfovibrio gracilis* DSM16080, *Desulfovibrio longus* DSM6739, *Desulfovibrio oxyclinae* DSM11498, and *Desulfovibrio* sp. BMSR_re-assembly.

The last persistent gene family, (DVU0434) Ech hydrogenase, subunit EchA, belongs to cytoplasmic hydrogenases. This gene was present in 55 genomes and was absent in the following eight genomes; *Oleidesulfovibrio alaskensis* G20 [32] *Desulfovibriotfgvc piger*, *Desulfovibrio piger* ERS1426881, *Desulfovibrio psychrotolerans*, *Desulfovibrio* sp. Bin1, *Desulfovibrio* sp. BMSR_re-assembly, *Desulfovibrio* sp. PG-178-WT-4, and *Desulfovibrio* sp. ZJ746.

#### 3.4.2. Shell Gene Family

Shell partition of the pangenome is the most dynamic part of gene families, and plays an important role in the evolution of genomes. A total of 17 gene families were non-uniformly distributed in the shell category of 63 SRB genomes. There were five gene families, distributed in 36–55 genomes, where DVU0430, *hydA*, *bfr*, *lytS*, and DVU2110 were present in 55, 43, 40, 38, and 36 genomes, respectively. These five genes encode for Ech hydrogenase, subunit EchE, Periplasmic [Fe] hydrogenase large subunit, Bacterioferritin, Histidine kinase, and L-lactate permease, respectively, and are part of significant metabolic pathways and mechanisms to sustain bacterial growth and energy generation. The remaining other 14 gene families have distributed between 8 and 25 genomes, and, therefore, this number is less than 50% of the total genomes used in the study.

#### 3.4.3. Cloud Gene Family

The cloud genome has only five gene families scattered in 1–9 genomes. It means these gene families are unique for a few strains, which may be useful for them in a specific habitat. *SodB*, which encodes for superoxide dismutase, is only found in *Desulfovibrio bizertensis* MKS_re-assembly. The genes *fdnG-3* and DVU3171 are present in DvH, however, it is not represented in the sunburst plot. This is because the gene families for the above-mentioned genes were different from the families selected for the heatmap generation. Moreover, the *gap-1* gene was found to be present in all these microorganisms: *Desulfovibrio bizertensis* MKS_re-assembly, *Desulfovibrio desulfuricans* ND132, *Desulfovibrio gracilis* DSM16080, *Desulfovibrio longus* DSM6739, *Desulfovibrio oxyclinae* DSM11498, and *Desulfovibrio* sp. BMSR_re-assembly (Supplementary Materials Table S5).

In summary, pangenome analysis can be employed to validate the text-mining outcomes regarding the genes related to sulfate reduction and biocorrosion. Moreover, it can be observed that the persistent gene families involved in lactate utilization, dissimilatory sulfate reduction, electron transport, hydrogen metabolism, metal transport, and sulfide formation were present in all SRB genomes. Previous studies have reported that the generation of sulfite (SO_3_^2−^), sulfides (S^2−^, HS^−^), organic acids (lactic acid, formic acid, pyruvic acid and acetic acid), and inorganic acids (H_2_SO_4_, H_2_SO_3_, HNO_2_) plays a crucial role in metal surface erosion and corrosion in biofilm. Other factors contributing to the corrosion process can be credited to the production of acetate creating an acidic environment due to the consumption of lactate or pyruvate by *D. desulfuricans*. Previous studies report that the production of hydrogen sulfide by SRB can act as a pitting (type of localized corrosion) activator, which can be further oxidized to thiosulfate (intermediate in sulfate reduction), which is an even more aggressive pitting activator [38,39,40,41,42,43]. This analysis could help us infer that by-products (acids) of substrate utilization and energy metabolism (sulfate reduction) are directly and indirectly involved in pitting metal surfaces and biocorrosion.

### 3.5. Metabolic Pathway Analysis

Proposed mechanisms for MIC by DvH include:

1. Enzyme secretion (e.g., hydrogenases) or secretion of active molecules (e.g., cytochrome, flavins).
2. Acid production (e.g., acetic acid and sulfuric acid) and other corrosive compounds such as ammonia and sulfide.
3. Formation of biofilm and EPS production that hold sites for metal ion binding [44].

#### 3.5.1. Energy Metabolism

The energy metabolism of DvH involves several genes coding for metal ion binding proteins to provide the cell with the required cofactors and oxidative stress tolerance. In addition, the use of sulfate as the terminal electron acceptor and hydrogen respiration further releases sulfides, thereby facilitating biocorrosion with metabolically driven cellular growth [14]. Hydrogenases play a significant role in the energy metabolism of *Desulfovibrio* spp., as they are involved in the catalysis of molecular hydrogen to protons and electrons. DvH is an ideal model to study the role of hydrogenases (Hases) in *Desulfovibrio* species, as the genome of DvH is composed of four periplasmic or membrane-bound Hases: two isoenzymes of [NiFe], one [NiFeSe], and one [FeFe], and two [NiFe] Hases facing the cytoplasm [45,46]. Pereira et al. elucidated that genes with the likes of Periplasmic facing—Ni-Fe Hydrogenases (*hynA1/B1*—DVU1922/DVU1921); Ni-Fe-Se Hydrogensases (*hysAB*—DVU1918/DVU1917) as well as Fe-Fe Hydrogenases (*hydA/B*—DVU1769/1770) exhibited a higher transcription expression on the basis of cation availability in the immediate environment of the bacteria, where they facilitate hydrogen uptake and utilization for energy metabolism [47]. Von Wolzogen Kuhr and van der Flugt postulated CDT, stating the function of hydrogenases and its involvement in MIC. According to CDT theory, the ability of SRB to either generate or consume molecular hydrogen by the activity of the hydrogenases is a critical factor in the deterioration of steel. Moreover, hydrogenase can withstand the biofilm stress for months, and can remain in an active state irrespective of the cell viability. Therefore, the mechanism of direct electron transfer between the metal surface and the enzyme can be established [44,48,49]. It has also been speculated that the presence of genes encoding cytoplasmic Ech hydrogenases (DVU0430 and DVU0434) results in compensational redundancy to hydrogen metabolism in addition to the periplasmic hydrogenases [50,51].

Throughout this metabolic process, the *fur* regulon (DVU0942) maintains the ferrous ion uptake and homoeostasis, while simultaneously responding to stress conditions of nitrate and alkaline stress upregulating the genes under its control. Parallel to hydrogen, formate is produced from pyruvate and lactate pathways, and absorbed formate is oxidised by formate dehydrogenases (DVU2812and DVU0588) [33,52,53]. The resultant electrons generated from hydrogen or formate oxidation are channeled towards the sulfate pathway via cytochrome complexes (DVU2524), producing hydrogen sulfide and facilitating corrosion [14].

#### 3.5.2. Electron Transport

**Figure 5:**
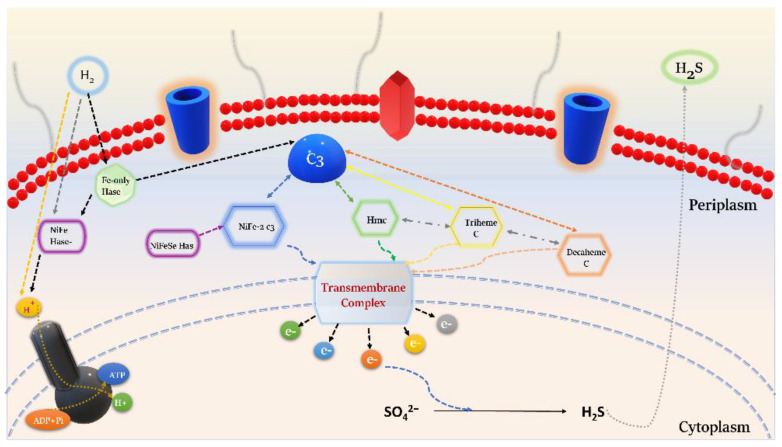
Diagrammatic description of a possible electron transport mechanism in *D. vulgaris* Hildenborough throughc-type cytochrome network and the associated periplasmic hydrogenases. The hydrogen oxidation generates electrons that are transferred into the c-type cytochrome via membrane-bound electron carriers, facilitating the reduction of sulfate to hydrogen sulfide.

SRB has intricate electron transport chains (ETC) that permit sulfate reduction by oxidation of either molecular hydrogen or organic compounds [44]. The initial step in the ETC process is the oxidation of hydrogen in the periplasm. The periplasmic hydrogenases metabolize H_2_ and facilitate its diffusion to the periplasm, where molecular hydrogen splits into electrons and protons. DVU3171 (tetrahemic cytochrome c3) is considered the primary electron acceptor from the oxidation of periplasmic hydrogen [54]. Now, the protons generate a proton-motive force for the production of ATP. Simultaneously, the cytochrome c3 network and various transmembrane complexes cycle back the electrons to the cytoplasm for sulphate reduction and other metabolic processes (Figure 5). The genome analysis of DvH also indicated the presence of alternate c3-type cytochromes (DVU2524 and DVU2809). DVU2524 could act as an electron acceptor for *hynAB* (DVU1921/22), whereas DVU2809 is part of the formate dehydrogenase operon, indicating that this tetraheme cytochrome c3 can accept electrons evolving from the oxidation of formate. A vast network of interlinked hemes implies intermolecular electron transfer amongst the various c-type cytochromes [14,55,56]. Heidelberg et al., 2004 also proposed a similar electron transfer mechanism in their study with *D. vulgaris* Hildenborough, suggesting this network-type to act as the connecting bridge for multiple periplasmic enzymes, including hydrogenases [14].

In 1995, Kloeke et al. also suggested a new model for the MIC of mild steel by DvH. They isolated a high molecular weight cytochrome (Hmc) graved in the outer membrane of DvH and indicated it as the “entry point” of the electrons and the redox partner of a [Fe]-Hase located in the periplasm of the bacteria [57,58]. The electron transfer mechanisms of hydrogenase/cytochrome can provide knowledge of a cell’s energy resources, especially when the cell is compelled to grow in a limited electron donor or acceptor medium. This information might be pertinent to environments where the microorganism can utilize any available metallic compounds, influencing biocorrosion activities such as those occurring in metal pipes or heat exchangers [59].

#### 3.5.3. Sulfur Metabolism

**Figure 6:**
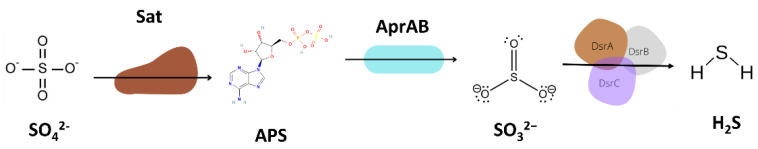
Summary of dissimilatory sulfate reduction pathway in SRB. Activation of sulfate to APS is carried out by the enzyme *sat* and further reduced by *AprAB* to sulfite. The final reduction of sulfite to hydrogen sulfide (H_2_S) is derived by the enzyme *DsrABC*.

Among microbes, the assimilatory sulfate reduction process is most prevalent, whereas dissimilatory sulfate reduction is observed only in limited microbial communities, including SRB. The first step in the dissimilatory sulfate reduction pathway is carried out by sulfate adenyl transferase (*sat*), which produces Adenosine-5′-phosphosulfate (APS) by the activation of sulfate, and, subsequently, reducing APS to sulfite by *AprAB*. Next, sulfite is partially reduced to hydrogen sulfide (H_2_S) by *DsrAB* (Dissimilatory sulfite reductase). Finally, *DsrC* carries out the final sulfite reduction to H_2_S (Figure 6) [60].

SRB utilize sulfate as the final electron acceptor for growth, with the instantaneous conversion to hydrogen sulfide [61]. The biological production of sulfide is of particular concern because H_2_S is a corrosive metabolite associated with oil souring and severe corrosion of metals in a water system [62]. In addition, H_2_S reacts with metal surfaces to produce metal sulfides (e.g., FeS). FeS induces a local decrease in pH by forming a thin film on the metal surface, which is highly unstable in nature., This elevates the cracking of the passive film, giving rise to highly active corrosion cells between the FeS acting as the cathode and the steel surface acting as anode [23,63]. Oxidation–reduction reactions of sulfur and sulfur compounds mediated by cells within biofilms are crucial mechanisms contributing to biocorrosion. Biofilm matrix might trap sulfur compounds containing sulfides, bisulfides, hydrogen sulfide (H_2_S), thiosulfates, and sulfuric acid (H_2_SO_4_). Moreover, H_2_S and H_2_SO_4_ can travel in gaseous and waterborne phases before reacting with materials [64].

*Desulfovibrio* species are ubiquitous in anoxic niches such as soils, sediments, and waters, where organic acids can be utilized as electron donors to carry out sulfate reduction. However, incompletely oxidizing sulfate reducers such as *D. vulgaris* produces acetic acid as a final product of organic acid oxidation, followed by the generation of electrons that are utilized for sulfate reduction. The ability of *D. vulgaris* to use other electron donors for sulfate reduction indicates the organism’s capability to detect a wide range of environmental cues. This implies the existence of a large number of the two-component signaling systems in the *D. vulgaris* genome which is marked by the presence of a few dozens of sensory histidine kinases and response regulators [65,66]. It is important to realize that the production of metabolic by-products by SRB is not enough evidence for their contribution to the corrosion process. However, the bacterial metabolic products can often promote new electrochemical reactions between the metal surface and the biofilm [27]. This attributes to the fact that the presence of biofilm on the metal surface is a crucial factor when considering MIC by SRB.

#### 3.5.4. Biofilm Formation

New electrochemical reaction pathways are established by the presence of biofilms on a metal surface. Biofilm enhances the rate of metal corrosion by stimulating responses which are usually unfavorable in the absence of microorganisms; such corrosion reactions can diminish the integrity of the metal surface [67]. Cell adhesion proteins (DVU2667) play an essential role in cell attachment and MIC. The formation of biofilm is preceded by the planktonic cells attachment to the metal surface, resulting in the production of EPS [68]. The GGDEF domain protein (DVU3106) extracted during text-mining has been implicated as a signaling factor promoting the switch of planktonic cells to the biofilm [69]. In addition, the DVU0670 and DVU0281 obtained were found to encode for proteins involved in EPS production [70]. One of the critical properties of SRB biofilm is the capability to influence corrosion by binding its EPS with metal ions [71]. The exopolymer matrix forms a protective layer, giving microbes resistance to antibiotics and facilitating horizontal gene transfer, making biofilm cells adapt to harsh environmental conditions [72]. The capability of EPS to capture metal ions and form complexes depends on the species of bacteria and the class of bound metal ion [73]. Additionally, Chan et al. reported the role of EPS alone as a metal corrosion agent. They clearly demonstrated the involvement of EPS in influencing MIC by examining metal corrosion in media comprising no EPS (control) and 1% EPS, which supported the idea that EPS can trap metal ions leading to metal corrosion [74,75]. These findings corroborate with the GO term analysis of the gene set, identifying metal ion binding as a significantly enriched GO term (*p*-value < 0.05) (Figure 2).

Biofilm formation requires stress response regulators, and a change in gene expression levels is triggered under different stress conditions that support the survival of the microbes. The environmental conditions under which biofilm formation takes place are of great importance as the control of the gene expression regulators involved in stress response greatly depends on the growth conditions of the microbe. Therefore, it can be suggested that the relative expression of various stress-related genes and proteins are dynamic in nature [3]. For instance, in DvH, the upregulation of *hcp* (DVU2013) and *nhac-2* (DVU3108) was observed when the cells were grown under nitrite and alkalinity stress, respectively [56]. Moreover, it was reported that *D. vulgaris* planktonic cells had fewer transcripts for the stress response protein superoxide dismutase (*sodB*, DVU2410) in comparison to biofilm [76].

The presence of a biofilm matrix on a metal surface creates anoxic compartments in which SRB multiply, forming localized corrosion pits and holes. In addition, direct contact with the metal surface and the outer membrane proteins of bacteria (such as cytochromes and hydrogenases) or electroconductive nanowires are established within the biofilm matrix [77]. Genes associated with flagellar biosynthesis, pilus assembly, EPS (DVU0670) synthesis and transport (DVU0117, DVU1892) were correlated with the biofilm formation. The cells in biofilm may induce corrosion either by the production of corrosive metabolites or by direct oxidation/reduction of metal ions, which leads to the disintegration of the passivating films. Moreover, evidence has emerged that two-component signal transduction systems are involved in forming biofilms, including a hybrid type kinase (DVU3026) that can take part in signal transduction, which is an integral part of cell–cell communication or differentiation [53]. Thus, proteins that participate in biofilm formation on the metal surface and contribute to the corrosion process should be considered to examine for MIC [78,79,80].

#### 3.5.5. Stress Response

Bacteria are exposed to various stresses in the natural environments. Thus, a variety of highly controlled adaptive responses are observed when the bacteria are exposed to several stresses, such as oxidative/nitrosative stress, nutrient starvation/limitation, ribosome disruption (ribosomal stress), and physical stress (membrane damage, heat and cold stress) which can lead to the formation of biofilm as a response to these stresses [81]. *Desulfovibrio* species in natural environments are often found close to oxic habitats such as soil, water, and the human oral cavity. Their survival in such environments requires a mechanism enabling resistance to oxidative stress. Data mining uncovered the existence of stress protein homologs in several SRB. Particularly, protein homologs participating in oxidative stress defense, such as superoxide dismutase (*sodB*), bacterioferritins (*bfr*), thioredoxins (*trx*), *hcp* and pyruvate–ferredoxin oxidoreductase *(poR*), were found in *Desulfovibrio* species [82].

Essential metabolic proteins are inactivated by releasing their metal centers when exposed to oxidative stress. Additionally, activation of the Fenton reaction results in reactive oxygen species (ROS) generation, which requires free metals, such as iron, as a source for its simultaneous production. This can be circumvented by storing iron in specialized proteins. To deal with iron bioavailability and simultaneously decrease ROS production, bacteria use a ferritin (Ft) family protein, which stores a substantial amount of Fe^2+^ ions in a form of ferric oxide mineral. The members of the ferritin family can be categorized into three major classes: *DPSs* (DNA-binding proteins from starved cells), ferritins (*Fts*), and bacterioferritins (*Bfrs*). The fundamental role of ferritins is to provide the cells with the required iron. For example, in anaerobic organisms, ferritins and bacterioferritins (DVU1397) play a role in the oxidative stress resistance mechanism in *D. vulgaris* by consuming dioxygen through the ferroxidase reaction [83,84,85].

Furthermore, Soldano et al. (2020) described the role of *bfr* in biofilm development. They postulated that sufficient iron is needed for the development of biofilm in *Pseudomonas aeruginosa*, and blocking the bacterioferritin–ferredoxin complex can lead to irreversible accumulation of unusable iron in *Bfr*, leading to acute cytosolic iron limitation. Therefore, deficiency of cytosolic iron can be observed in the planktonic and biofilm-embedded cells when the *Bfr-Bfd* complex is blocked, which results in poor biofilm development even in culture conditions with adequate iron [86,87]. Moreover, Qi et al. suggested that gene DVU2571 encoding for ferrous iron transport protein B *(feoB)* is required under high iron concentrations to maintain the integrity of *D. vulgaris* biofilm [88]. Other small molecules known to help combat oxidative stress are *trx-B* (DVU1838), which maintains an anaerobic intracellular environment by either scavenging the ROS or by reduction of the protein disulfide bonds created by several oxidants. Moreover, *trx-B* plays a role in cellular defense by regenerating the proteins damaged by oxidative stress, by modulating the movement of redox stressors. Thioredoxins can also behave as donors of hydrogen, detoxifying enzymes involved in oxidative stress response. For example, in (SRB), the *poR* (DVU3025), plays a vital role in anaerobic metabolism, and is activated by trx after an exposure to oxidative stress [37,89,90]. Oh, et al. (2016), in their study with a microaerophilic, *Campylobacter jejuni*, described the enhanced biofilm formation of *C. jejuni* under aerobic conditions. They also revealed that oxidative stress has a significant role in the development of *C. jejuni* biofilm under microaerophilic conditions [91]. Similarly, Bade et al. demonstrated the capacity of SRB to form biofilm and survive in the presence of oxygen, causing severe corrosion in drinking water systems [92]. These results suggest the involvement of stress response genes in biofilm formation and MIC.

## 4. Conclusions

Currently, different methods are being explored to prevent and treat MIC. Consequently, understanding the microbial corrosion mechanism is essential to improving corrosion inhibition results. Generally, the biocorrosion process is controlled by the metabolic activities of the sulfate-reducing bacteria, their biofilm structure, and electron transfer mechanisms. The role of SRB in MIC can be further attributed to the presence of metal in sulfate-containing anaerobic conditions, which can exacerbate the biocorrosion reactions. Moreover, anaerobically eroded metal is characterized universally by the existence of SRB and its corrosion products, e.g., metal sulfides such as FeS. Hence, SRB, as the main culprit in anoxic-metal corrosion, has been reported by numerous studies, demonstrating the role of sulfur metabolism and EPS formation as metal corrosion-enhancing pathways. Additionally, SRB can cause metal corrosion by directly utilizing the metal by forming a direct electron transfer mechanism in anoxic environments. Therefore, the methods involved in MIC cannot be narrowed down to one single process, but rather are a conglomeration of multiple regulatory responses. Nevertheless, with text-mining, researchers are provided with the benefit of directing their research precisely to the gene/protein level, making the screening process much easier and less time-consuming. Through pangenome and cluster analysis, it has been observed that the genes involved in sulfur metabolism (e.g., *dsrABC* and *sat*), hydrogen cycling and energy metabolism (e.g., hydrogenases and c-type cytochromes), and biofilm formation (e.g., DVU0670 and DVU0281), have been indicated to play an active role in MIC. However, experimental validation is required to elucidate the involvement of these genes in MIC. Our current findings provide a framework to further investigate the biocorrosion mechanism in sulfate-reducing bacteria. Integrated gene knockout studies and metabolomic analysis of *D. vulgaris* are crucial to substantiate the role of identified genes by text-mining.

## Data Availability

Not applicable.

## Supplementary Materials

The following supporting information can be downloaded at: https://www.mdpi.com/article/10.3390/microorganisms11010119/s1. Table S1: Gene list from GenNer; Table S2: Manually curated gene list; Table S3: Genes involved in biocorrosion; Table S4: Pangenome presence/absence matrix; Table S5: Genes absent in the sunburst plot.
